# Proteomic Identification of Genes Associated with Maize Grain-Filling Rate

**DOI:** 10.1371/journal.pone.0059353

**Published:** 2013-03-19

**Authors:** Xining Jin, Zhiyuan Fu, Dong Ding, Weihua Li, Zonghua Liu, Jihua Tang

**Affiliations:** College of Agronomy, Key Laboratory of Physiological Ecology and Genetic Improvement of Food Crops in Henan Province, Henan Agricultural University, Zhengzhou, China; University of Nottingham, United Kingdom

## Abstract

Grain filling during the linear phase contributes most of the dry matter accumulated in the maize kernel, which in turn determines the final grain yield. Endosperms and embryos of three elite maize hybrids (Zhengdan 958, Nongda 108, and Pioneer 335) were sampled 17, 22, 25, and 28 days after pollination, during the linear phase of grain filling, for proteomic analysis to explore the regulatory factors critical for grain filling rate. In total, 39 and 43 protein spots that showed more than 2-fold changes in abundance at *P*<0.01 between any two sampling stages in the endosperm and embryo were analyzed by protein mass spectrometry. The changing patterns in expression index of these proteins in the endosperm were evenly distributed, whereas up-regulation patterns predominated (74%) in the embryo. Functional analysis revealed that metabolism was the largest category, represented by nine proteins in the endosperm and 12 proteins in the embryo, of the proteins that significantly changed in abundance. Glycolysis, a critical process both for glucose conversion into pyruvate and for release of free energy and reducing power, and proteins related to redox homeostasis were emphasized in the endosperm. Additionally, lipid, nitrogen, and inositol metabolism related to fatty acid biosynthesis and late embryogenesis abundant proteins were emphasized in the embryo. One protein related to cellular redox equilibrium, which showed a more than 50-fold change in abundance and was co-localized with a quantitative trait locus for grain yield on chromosome 1, was further investigated by transcriptional profile implying consistent expression pattern with protein accumulation. The present results provide a first step towards elucidation of the gene network responsible for regulation of grain filling in maize.

## Introduction

Maize (*Zea mays* L.) is one of the most important cereal crops cultivated worldwide, and serves as a source of food, feed and fuel. Among the three key components of grain yield in maize, kernel weight is crucial. The embryo, which requires the coordinated development of multiple tissues and cell types, and the endosperm, a starch-rich tissue that supports the embryo during germination, are the main contributors to final kernel weight. Grain productivity is the result of grain-filling rate and duration, which contribute greatly to the accumulation and distribution of dry matter in the kernel [1]. During the effective grain-filling period, the grain-filling rate is positively correlated with grain weight [2]–[4]. Thus, improvement of the grain-filling rate is an important objective of breeders in order to achieve high yields, especially under stress conditions [5]–[7].

In maize, accumulation of kernel dry matter influenced by grain filling rate is a continuous process that can be divided into three characteristic stages [8]. The first stage occurs from fertilization to 12 days after pollination (DAP). During this lag phase, the cells expand rapidly in number and size, which determines the ‘storeroom’ for accumulation of photosynthate [9], [10]. The cell number might have a greater impact on final grain yield than cell size [11]–[15]. As the primary source of maize kernel dry matter [16], carbohydrates from leaf photosynthates are rapidly transported to the developing grain during the linear phase of grain filling. The linear phase ranges from 12 to 40 DAP with the most rapid grain filling occurring between 21 and 25 DAP. Notably, the conversion of imported sucrose and amino acids into starch and storage proteins during this stage can account for about 90% of kernel total dry matter [17], [18]. The final phase is the ‘desiccation–maturation’ stage, which occurs from 40 to 70 DAP. In this stage, a decreasing grain-filling rate culminates in physiological maturity and black-layer formation, and is related to seed dormancy [18], [19]. The duration of each stage is variable depending on genetic background and environmental temperature [4].

Given that the linear phase has important implications for final maize grain yield, its underlying molecular mechanisms have been studied at different levels. Liu et al. evaluated the grain-filling rate of a set of 203 recombinant inbred lines at two locations over two years using 217 simple sequence repeat (SSR) markers and identified six stable quantitative trait loci (QTL) for grain-filling rate [20]. Méchin et al. established a proteome reference map for an INRA F_2_ maize inbred line of the endosperm at 14 DAP by means of two-dimensional (2-D) gel electrophoresis and protein identification with liquid chromatography–mass spectrometry (MS) [17]. Prioul et al. further investigated the endosperm transcriptome of this F_2_ inbred line with cDNA libraries constructed at the lag phase (10 DAP), onset of carbohydrate storage (14 DAP), and maximum starch accumulation rate (21 DAP). The 10 DAP transcriptome and 14 DAP proteome showed good agreement [18]. Although some QTL and important functional protein classes were identified during the grain-filling stage, derivation of the endosperm proteome from a single inbred line is inadequate to understand the molecular mechanisms and crucial regulatory factors responsible for grain filling. Hybrid maize genotypes, which show a grain-filling rate distinct from that of inbred lines, predominate in terms of cultivated area and grain yield worldwide compared with inbred lines. Thus, dissection of the molecular mechanism of grain-filling rate in hybrids rather than inbred lines is more important for improvement of grain yield by genetic manipulation in maize. The embryo, which develops from the fertilized egg and thus is genomically distinct from the maternal tissue-derived endosperm, predominantly accumulates fatty acids and also contributes importantly to kernel weight. However, in cereals proteomic analysis of the embryo during the linear phase has been reported only in rice [21]. Thus, to elucidate the molecular mechanisms responsible for grain yield in maize, integration of genomic and proteomic assays for endosperm and embryo of hybrid maize should be undertaken synchronously.

As an important exploratory technique at the translational level, proteomic technology, which combines the resolution of 2-D gel electrophoresis with the sensitivity of MS, was used in the present study to dissect the specific proteins that accumulate in the endosperm and embryo during the linear phase of grain filling in three elite hybrids of maize (Zhengdan 958, Nongda 108, and Pioneer 335), which are widely cultivated in China. The main objectives were: (1) to obtain comparative information on proteins differentially accumulated between the endosperm and embryo during the linear phase of grain filling; and (2) to identify potential candidate genes that influence grain filling in maize.

## Results

### Analysis of Grain-filling Rate

**Figure 1:**
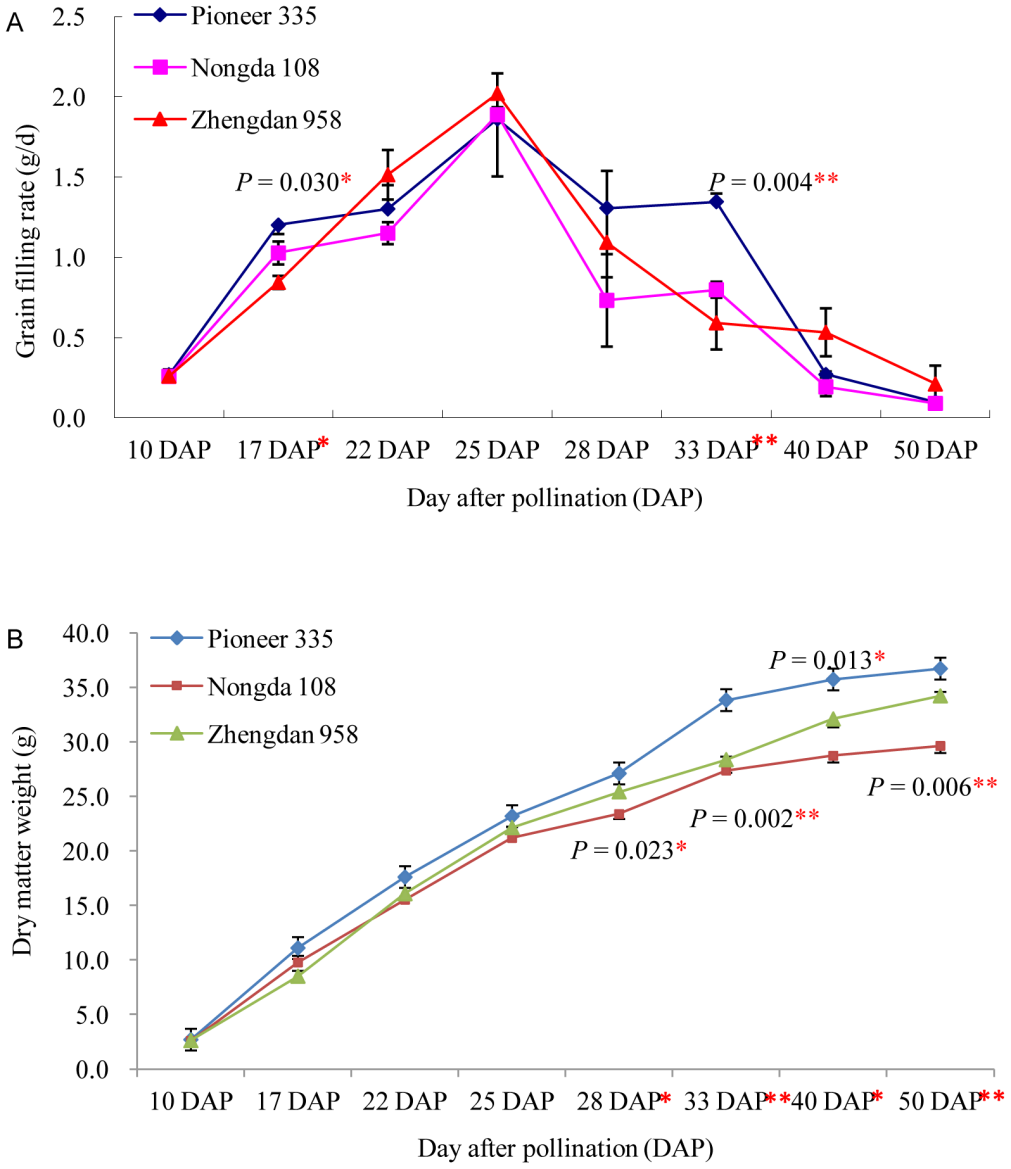
Grain-filling rate of the tested maize hybrids from 10 to 50 DAP.

During the entire period of dry matter accumulation (10 to 50 DAP), the three elite hybrids showed significant differences in final grain dry weight (*P* = 0.006) (Figure 1B) and grain-filling rate (*P* = 0.004) (Figure 1A). In addition, the grain-filling rate at 17 and 33 DAP (Figure 1A), and grain dry weight at 28 DAP (Figure 1B) also showed significant differences between the three hybrids. Grain-filling rate dynamics was different for the three tested hybrids but with the same peak at 25 DAP (Figure 1A). Zhengdan 958 showed a steep increase before 25 DAP and a more moderate decrease after 28 DAP in grain-filling rate, which was significantly distinguish from that of Nongda 108 and Pioneer 335 with distinct grain-filling rate from 25 to 40 DAP (Figure 1A). These results implied that dry matter accumulation might mainly derive from rapid grain filling before 25 DAP and a relatively uniform decline after 25 DAP in Zhengdan 958; in contrast, dry matter accumulation was mainly attributable to the comparatively high grain-filling rate after 25 DAP, especially between 25 and 40 DAP, in Pioneer 335.

### Identification of Differentially Expressed Proteins

**Figure 2:**
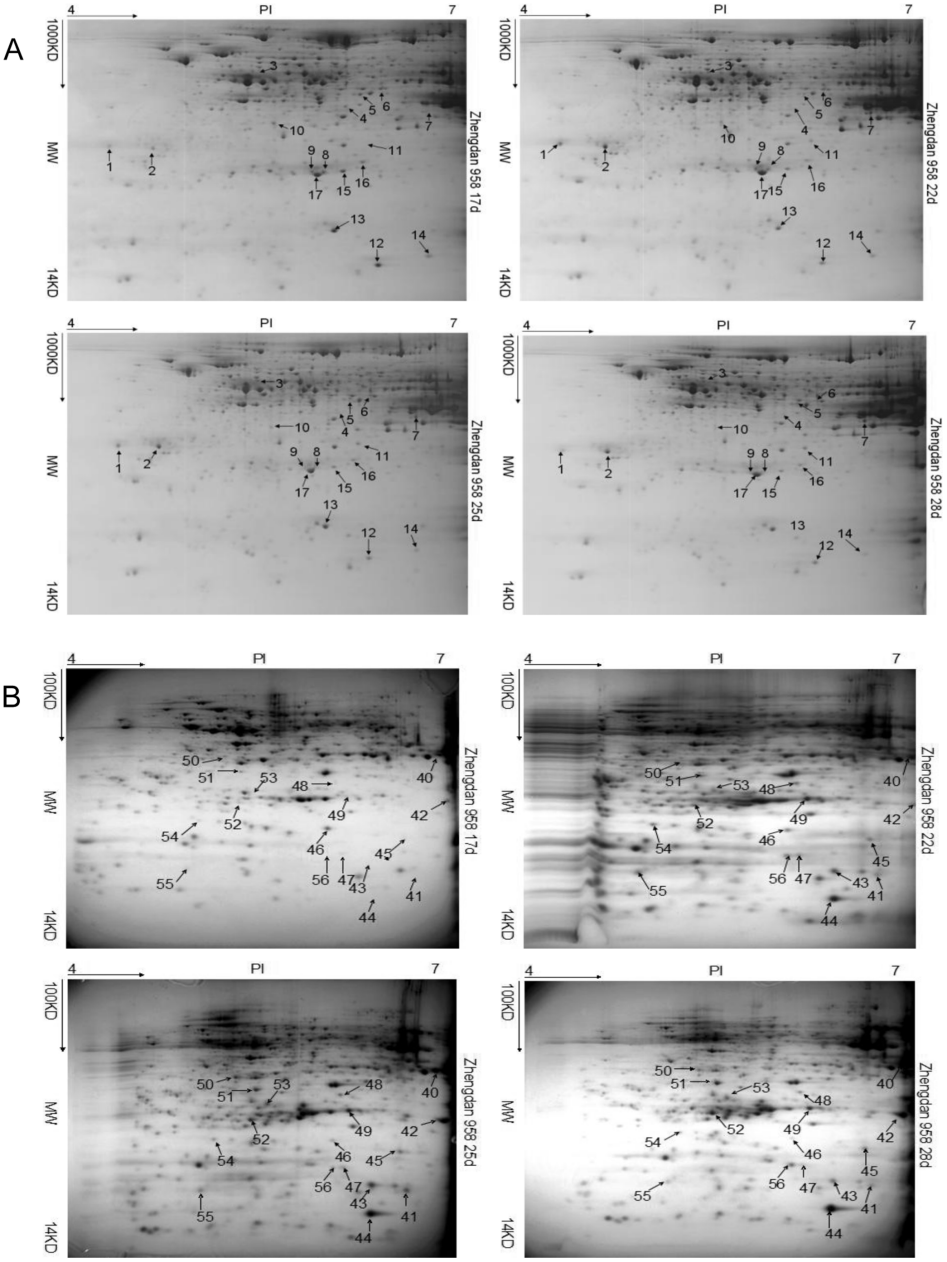
Representative 2-D maps. Maps from left to right represent expressed proteins at 17, 22, 25, and 28 days after pollination in the endosperm (A) and embryo (B) of Zhengdan 958, respectively. Differentially expressed protein spots at any two stages with more than 2-fold changes in expression are indicated by arrows with numeral.

The endosperm and embryo of grains were sampled at 17, 22, 25, and 28 DAP for proteomic analysis within a linear gradient of pH 4–7 on the basis of the observed changes in grain-filling rate. At these respective sampling stages, 568, 655, 583, and 507 protein spots in the endosperm and 638, 523, 614, and 463 protein spots in the embryo were identified for Zhengdan 958; 565, 437, 527, and 498 protein spots in the endosperm and 691, 704, 699, and 345 protein spots in the embryo were identified for Nongda 108; and 439, 501, 426, and 383 protein spots in the endosperm and 600, 707, 675, and 802 protein spots in the embryo were identified for Pioneer 335 (Table 1). Compared with the embryo, a greater number of protein spots were identified at 25 DAP and fewer protein spots were identified at 17 DAP for each genotype in the endosperm. Furthermore, analysis of variance (ANOVA) and *t*-test analyses revealed that 281 and 183, 278 and 209, and 244 and 193 protein spots showed significantly differential expression in the endosperm and embryo of Zhengdan 958, Nongda 108, and Pioneer 335, respectively, at the four sampling stages (*P*<0.01) (Table 1). Among these spots, 39 spots in the endosperm (17 for Zhengdan 958, 10 for Nongda 108, and 12 for Pioneer 335) and 43 spots in the embryo (17 for Zhengdan 958, 14 for Nongda 108, and 12 for Pioneer 335), all of which showed greater than 2-fold changes in abundance at *P*<0.01, were excised and analyzed by MS for protein identification (Figure 2; Figure S1).

**Table 1:** Number of differentially expressed protein spots in endosperm and embryo of the three maize hybrids at different sampling times during grain development.

Tissue	Material name	Total identified protein spot				Differentially expressed protein spot
		17 DAP	22 DAP	25 DAP	28 DAP	
Endosperm	Zhengdan 958	586	655	583	507	281
	Nongda 108	565	437	527	498	278
	Pioneer 335	439	501	426	383	244
Embryo	Zhengdan 958	638	523	614	463	183
	Nongda 108	691	704	699	345	209
	Pioneer 335	600	707	675	802	193

### Changing Pattern and Functional Analysis of Differentially Expressed Proteins

The differentially expressed proteins in the endosperm (39 protein spots) and embryo (43 protein spots) were revealed in seven groups (Figure S2). Compared with the endosperm, continuously up-regulated proteins formed the largest group (group I) represented by 13 out of 39 proteins in the embryo. Proteins with the accumulation peak at 25 DAP (Group III) were also frequent in the embryo, which was represented by 12 out of 39 proteins. In contrast, expression patterns in the endosperm were more uniformly distributed over the sampling period (Table S1).

**Figure 3:**
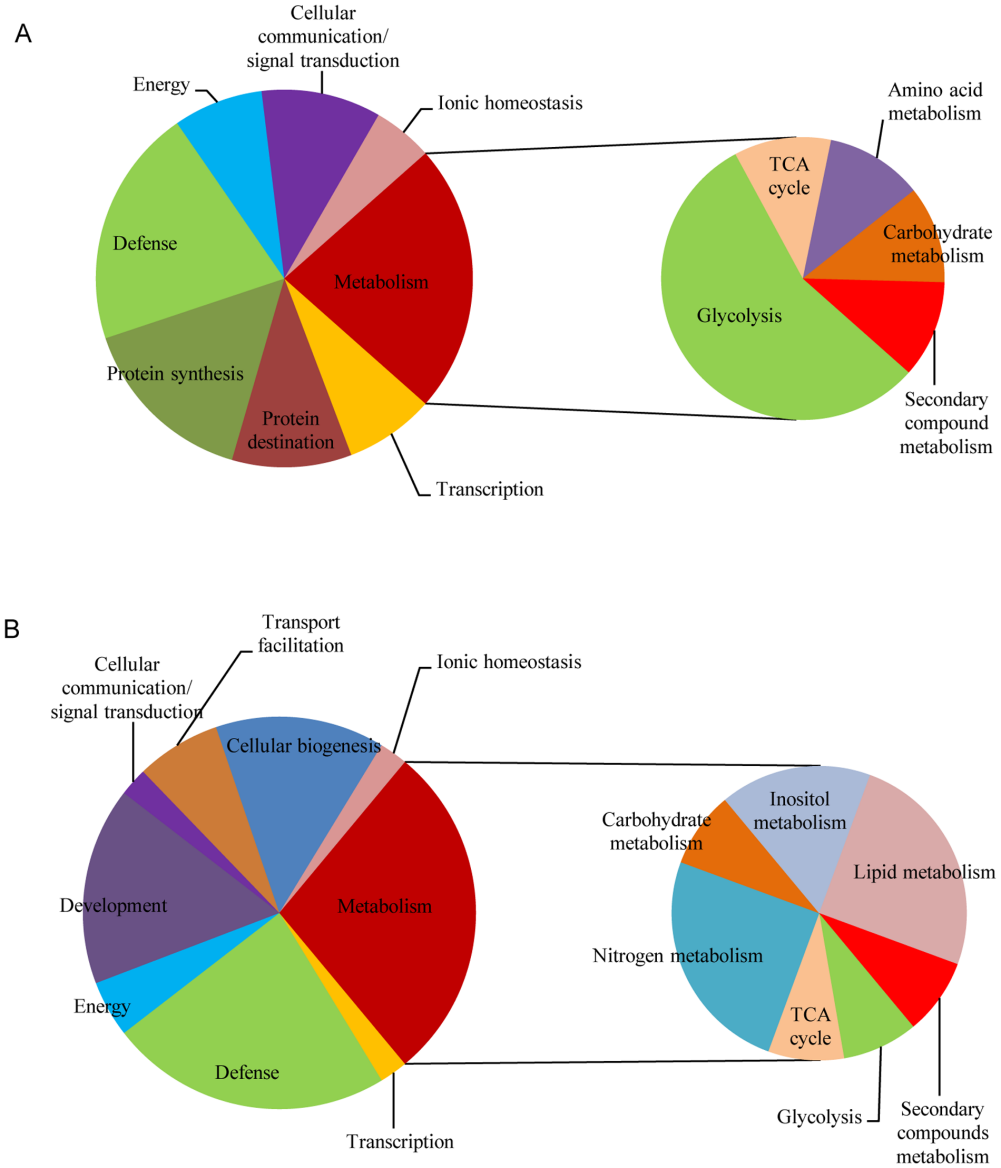
Functional category of the labeled proteins in 2-D maps of the three maize hybrids studied. A: the protein category in the endosperm; B: the protein category in the embryo.

On the basis of functional annotations, seven categories of proteins in the endosperm (Figure 3A) and eight categories in the embryo were screened (Figure 3B). The largest functional category was metabolism, which was represented by nine and twelve proteins in the endosperm and embryo, respectively. This category was further subdivided into five and seven subcategories in order to analyze in more detail dynamic changes in metabolism for the endosperm and embryo, respectively. Proteins involved in glycolysis (five proteins) in the endosperm, and proteins involved in lipid metabolism (three proteins) and nitrogen metabolism (three proteins) in the embryo were the predominant subcategories of the metabolism category. Defense proteins, the second-largest category, were represented by eight proteins in the endosperm and ten proteins in the embryo. In the endosperm, the remaining proteins were classified as protein synthesis (six proteins), protein destination (four proteins), cellular communication proteins (four proteins), energy proteins (three proteins), and ionic homeostasis proteins (two proteins). Proteins associated with development (seven proteins), cellular biogenesis (six proteins), transport facilitation proteins (three proteins), energy proteins (two proteins), cellular communication proteins (one protein) and ionic homeostasis proteins (one protein) were the remaining categories in the embryo.

**Figure 4:**
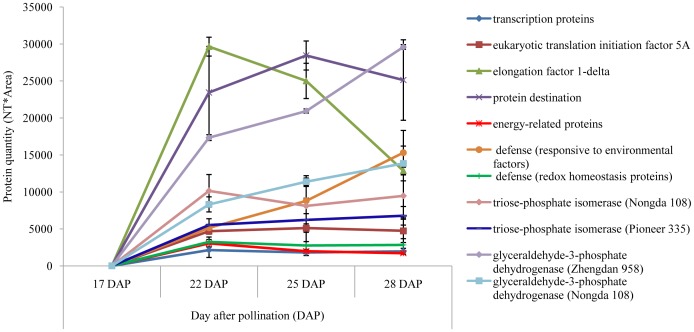
Plot figure for represented differentially expressed protein spots in endosperm with relative intensities.

In the endosperm, transcription proteins and eukaryotic translation initiation factor 5A (Table 2) were down-regulated. Elongation factor 1-delta was up-regulated in abundance during grain filling. The expression changes of transcription, translation initiation and elongation proteins during the linear phase of grain filling were in accordance with the protein synthesis process (Figure 4). Proteins involved in protein destination were up-regulated. Energy-related proteins that showed continuous down-regulation were identified only in Zhengdan 958. In the defense category, proteins responsive to environmental factors were up-regulated but the reverse applied to redox homeostasis proteins. In the largest metabolism category, two major glycolysis proteins were identified, namely triose-phosphate isomerase in Nongda 108 (spot no. 27) and Pioneer 335 (no. 39), showing a differential expression pattern, and glyceraldehyde-3-phosphate dehydrogenase in Zhengdan 958 (no. 7) and Nongda 108 (no. 25) showing an identical up-regulation expression pattern (Figure 4).

**Table 2:** Annotation of differentially expressed proteins identified in the endosperm and embryo of the three maize hybrids during the linear phase of grain filling.

Hybrid	Spot no.^a^	Accession no.^b^	Protein name [*Zea mays*]^c^	Function^d^	*M*_r_ gel/*M*_r_ predicted^e^	pI gel/pI predicted^f^	Peptide count^g^	Total ion score^h^	Total ion score C.I. (%)^i^	Score^j^	Coverage (%)^k^	*P*-value^l^
Zhengdan 958	1	gi|226503896	elongation factor 1-delta 1	Protein synthesis	40400/24951	4.2/4.4	1	64.54	100	65	4	8.00E−65
	2	gi|257673482	general regulatory factor1	Protein destination	40100/29757	4.5/4.8	3	91.88	100	92	18	2.20E−133
	3	gi|254256262	pyruvate, orthophosphate dikinase1	C metabolism	78700/106168	5.3/6.1	3	67.51	100	68	3	0.00E+00
	4	gi|162459370	glutamine synthetase2	Energy	57200/40462	5.8/5.6	10	108	100	169	38	3.50E−169
	5	gi|195634941	sarcosine oxidase	Amino acid metabolism	62500/44129	6.0/5.8	4	97.39	100	97	11	6.00E−115
	6	gi|219895497	IAA-glu synthetase	Secondary compound metabolism	63100/50135	6.1/5.8	3	150.42	100	150	7	1.40E−81
	7	gi|194701792	cytosolic glyceroldehyde-3-phosphate dehydrogenase GAPC2	Glycolysis	52300/36632	6.2/6.4	5	151.97	100	152	21	1.20E−146
	8	gi|257696234	APx1 - Cytosolic Ascorbate Peroxidase	Defense	31100/33693	5.8/8.9	2	50.64	99.97	51	8	3.30E−114
	9	gi|257696218	APx1 - Cytosolic Ascorbate Peroxidase	Defense	31100/27509	5.6/5.7	10	169	100	169	59	2.30E−113
	10	gi|195639126	fructokinase-2	Energy	52600/35954	5.5/5.3	4	158.7	100	159	12	1.30E−121
	11	gi|227308398	sorbitol dehydrogenase homolog1	Ionic homeostasis	40000/39747	6.0/6.3	4	146.44	100	146	14	1.80E−138
	12	gi|219906911	glycine-rich RNA binding protein	Protein synthesis	12100/15532	6.0/6.1	2	46.69	99.94	47	15	7.20E−32
	13	gi|195618512	eukaryotic translation initiation factor 5A	Protein synthesis	18300/17713	5.8/5.6	2	43.39	99.83	43	13	7.30E−71
	14	gi|195622856	nucleoside diphosphate kinase 1	Energy	13400/16530	6.1/6.3	2	79.84	100	80	16	4.80E−67
	15	gi|257643482	APx1 - Cytosolic Ascorbate Peroxidase	Defense	32100/39482	5.9/7.1	2	33.38	98.63	33	9	2.30E−113
	16	gi|238013894	uncharacterized LOC100502283	Cellular communication/signal transduction	34500/25665	6.0/5.9	3	74.98	100	75	19	1.40E−85
	17	gi|259703147	triosephosphate isomerase	Glycolysis	30900/27236	5.7/5.5	4	128.27	100	128	18	5.30E−100
Nongda 108	18	gi|195605696	elongation factor 1-delta 1	Protein synthesis	4040/24977	4.2/4.4	2	46.69	99.89	47	6	3.00E−65
	19	gi|262359935	general regulatory factor1	Protein destination	40100/29757	4.5/4.8	4	75.15	100	75	22	2.20E−133
	20	gi|37932483	glyoxalase I	Defense	41300/32450	5.8/5.6	3	90.49	100	90	11	1.10E−115
	21	gi|22190	protein b-32	Protein destination	45500/33467	6.0/6.0	3	147.48	100	147	12	5.90E−02
	22	gi|194708718	LOC100274593	Defense	19000/17815	5.6/5.7	4	75.3	99.66	75	34	2.10E−43
	23	gi|195605740	eukaryotic translation initiation factor 5A	Protein synthesis	18300/17713	5.8/5.6	5	51.62	99.98	52	13	7.30E−71
	24	gi|219888685	glycine-rich RNA binding protein	Protein synthesis	134000/14642	6.1/5.7	2	128	100	128	82	7.40E−32
	25	gi|194701792	cytosolic glyceroldehyde-3-phosphate dehydrogenase GAPC2	Glycolysis	52400/36632	6.5/6.4	7	72.8	100	73	17	1.20E−146
	26	gi|238013894	uncharacterized LOC100502283	Cellular communication/signal transduction	34500/25665	6.0/5.9	4	69.04	100	69	19	1.40E−85
	27	gi|195605636	triose-phosphate isomerase, cytosolic	Glycolysis	31000/27278	5.7/5.5	3	140.42	100	140	18	5.20E−100
Pioneer 335	28	gi|194699380	elongation factor 1-delta 1	Protein synthesis	40400/24951	4.2/4.4	4	77.24	100	77	10	8.00E−65
	29	gi|257710933	general regulatory factor1	Protein destination	40100/29724	4.5/4.8	2	61.28	100	61	18	2.00E−132
	30	gi|226496099	hypothetical protein LOC100274355	Cellular communication/signal transduction	62100/47785	6.0/6.0	3	53.37	99.98	53	6	2.90E−122
	31	gi|226498420	hypothetical protein LOC100273405	Ionic homeostasis	57200/48681	6.0/6.1	2	63.1	100	63	9	2.80E−181
	32	gi|257645318	malate dehydrogenase5	Tricarboxylic acid cycle	51300/35909	5.9/5.8	3	243	100	243	69	8.70E−157
	33	gi|257696218	APx1 - Cytosolic Ascorbate Peroxidase	Defense	32100/27509	5.9/5.7	2	204	100	204	63	2.40E−113
	34	gi|238013894	uncharacterized LOC100502283	Cellular communication/signal transduction	34500/25665	6.1/5.9	2	28.47	96.48	28	13	1.40E−85
	35	gi|194708718	LOC100274593	Defense	19100/17815	5.4/5.7	5	79.9	99.88	80	34	2.10E−43
	36	gi|195618512	eukaryotic translation initiation factor 5A	Protein synthesis	18300/17713	5.8/5.6	2	50.26	99.96	50	13	7.30E−71
	37	gi|195658559	trypsin/factor XIIA inhibitor precursor	Defense	12300/16861	5.9/8.1	2	108.04	100	108	10	1.50E−29
	38	gi|257743783	elongation factor 1−delta 1	Protein synthesis	13500/15532	6.0/6.1	2	35.6	99.07	36	28	7.20E−32
	39	gi|259703147	Hb	Glycolysis	30900/27236	5.7/5.5	3	109.28	100	109	13	5.30E−100
Zhengdan 958	40	gi|226507242	hypothetical protein LOC100274379	Cellular biogenesis	68000/38769	6.8/6.3	8	87.1	97.66	87	22	1.50E−49
	41	gi|194700850	cytosolic glyceroldehyde-3-phosphate dehydrogenase GAPC2	Glycolysis	22000/32076	6.6/6.4	7	91.52	100	92	9	1.20E−127
	42	gi|194706410	1-Cys peroxiredoxin antioxidant	Defense	42000/25126	6.9/6.4	10	136.49	100	132	47	5.80E−80
	43	gi|195607718	hypothetical protein	Defense	23000/47848	6.7/6.7	2	38.24	98.88	38	3	1.40E−09
	44	gi|1169520|sp|P46517.1|EMB5_MAI	RecName: Full = Late embryogenesis abundant protein EMB564	Development	18000/9677	6.3/6.6	2	42.8	98.91	108	43	1.10E−23
	45	gi|195642018	stress-inducible membrane pore protein	Transport facilitation	32000/17918	6.5/6.4	3	148.47	100	148	14	8.10E−26
	46	gi|195638850	glycine-rich protein 2b	Nucleotide metabolism	35000/20576	6.0/5.9	2	104.35	100	104	19	1.40E−35
	47	gi|195635073	hypothetical protein	Defense	27000/11830	6.1/5.1	3	64.3	95.75	64	55	8.40E−40
	48	gi|195659143	secreted protein	Transport facilitation	54000/27503	6.1/5.8	7	131.97	100	76	32	1.00E−66
	49	gi|195658029	lipoprotein	Cellular communication/signal transduction	47000/26539	6.2/5.9	1	63.74	99.85	64	5	2.80E−80
	50	gi|194707248	thiamine biosynthesis2	Nitrogen metabolism	64000/37454	5.3/5.6	1	42.3	99.81	42	3	2.90E−124
	51	gi|195658465	late embryogenesis abundant protein D-34	Development	57000/27274	5.5/5.4	3	83.45	100	83	16	4.30E−59
	52	gi|228310	globulin 2	Defense	43000/50234	5.5/6.2	2	92.08	100	92	6	4.60E−64
	53	gi|195606798	cupin family protein	Cellular biogenesis	50000/56775	5.6/6.1	2	33.19	98.68	33	5	1.90E−102
	54	gi|195638870	ATP synthase D chain, mitochondrial	Energy	36000/19914	5.2/5.2	8	128	100	128	61	1.00E−64
	55	gi|257708811	hypothetical protein(Sorghum bicolor)	Transport facilitation	22000/10891	5.1/5.0	2	90.81	100	91	25	2.40E−42
	56	gi|195658877	hypothetical protein	Cellular biogenesis	27000/13413	6.0/5.6	2	70.85	100	71	20	6.40E−10
Nongda 108	57	gi|162460575	1-Cys peroxiredoxin antioxidant	Defense	40000/25060	6.7/6.3	4	174	100	174	26	1.20E−79
	58	gi|194708200	uncharacterized LOC100274491	Inositol metabolism	81000/42380	6.0/5.2	2	54.92	99.99	55	7	2.30E−204
	59	gi|194707114	uncharacterized LOC100274264	Tricarboxylic acid cycle	62000/35491	6.1/8.2	2	50.01	99.97	50	11	3.20E−118
	60	gi|194707256	uncharacterized LOC100274292	Cellular biogenesis	53000/33565	6.1/6.0	4	72.39	100	72	21	4.90E−51
	61	gi|253786492	LOC100272498	Ionic homeostasis	52000/33151	6.1/5.8	3	126.48	100	126	13	1.00E−98
	62	gi|195659367	lipoprotein	Lipid metabolism	42000/26549	6.0/5.9	4	147.85	100	148	22	3.90E−81
	63	gi|195639126	fructokinase-2	Energy	59000/35954	5.4/5.3	5	172.21	100	172	20	1.30E−121
	64	gi|195658465	late embryogenesis abundant protein D-34	Development	51000/27274	5.2/5.4	4	89.55	100	90	26	4.30E−59
	65	gi|596078	thiamine biosynthetic enzyme	Nitrogen metabolism	58000/37308	5.1/5.2	2	67.5	100	68	9	2.80E−126
	66	gi|195653831	6-phosphogluconolactonase	C metabolism	46000/34874	5.0/7.7	3	117.15	100	117	12	9.20E−89
	67	gi|226502636	hypothetical protein LOC100276943	Lipid metabolism	28000/15621	5.3/12.3	1	65.59	100	66	8	9.30E−09
	68	gi|195658153	embryonic abundant protein 1	Development	18000/9663	6.1/6.6	2	74.19	100	74	27	8.40E−24
	69	gi|162464017	embryo specific protein5	Development	21000/11933	5.3/5.6	1	38.3	99.54	38	10	6.90E−29
	70	gi|257696216	ascorbate peroxidase	Defense	42000/27467	5.8/5.6	3	49.62	99.97	50	18	1.90E−113
Pioneer 335	71	gi|223973215	inositol-3-phosphate synthase-like	Inositol metabolism	80000/56278	6.3/5.8	2	81.25	100	81	4	3.20E−244
	72	gi|195607992	succinate semialdehyde dehydrogenase	Secondary compounds metabolism	75000/52875	6.0/5.7	3	46.53	99.93	47	10	2.60E−203
	73	gi|226533140	hypothetical protein LOC100274292	Cellular biogenesis	53000/33565	6.4/6.0	8	126	100	126	42	4.90E−51
	74	gi|239985530	thiamine biosynthetic enzyme precursor	Nitrogen metabolism	56000/37308	5.4/5.2	2	64.83	100	65	9	2.80E−126
	75	gi|195628018	enoyl-[acyl-carrier-protein] reductase [NADH]	Lipid metabolism	53000/39381	5.5/7.6	3	89.49	100	89	9	7.00E−123
	76	gi|195658465	late embryogenesis abundant protein D-34	Development	50000/27274	5.5/5.4	4	76.59	100	77	24	4.30E−59
	77	gi|257696218	APx1 - Cytosolic Ascorbate Peroxidase	Defense	43000/27509	6.1/5.7	4	57.78	99.99	58	26	2.40E−113
	78	gi|195658011	globulin-1 S allele precursor	Defense	38000/50275	5.8/6.2	2	93.61	100	94	6	6.80E−61
	79	gi|228310	globulin 2	Defense	38000/50234	5.7/6.2	2	90.86	100	91	6	4.60E−64
	80	gi|195658137	embryonic abundant protein 1	Development	20000/9663	6.4/6.6	2	67.71	100	68	27	8.40E−24
	81	gi|223945515	uncharacterized LOC100502218	Defense	20000/22859	4.6/5.7	5	69.1	98.59	69	30	3.20E−08
	82	gi|212722552	hypothetical protein LOC100193701	Cellular biogenesis	43000/26549	6.3/5.9	2	52.39	99.97	52	13	3.90E−81

a Number of identified differentially expressed protein spots on each 2-D map. Preferentially accumulated proteins: more than 2-fold changes; *t*-test or ANOVA: *P*<0.01 in three independent biological replicates.

b Accession number of each protein identified by MS.

c Identified proteins obtained from the NCBI *Zea mays* protein sequence database using the TurboSEQUEST algorithm.

d Functional annotation of each protein identified by MS.

e Molecular weight of protein on gel/predicted molecular weight of protein.

f pI of protein on gel/predicted pI of protein.

g Number of peptides matching the corresponding protein.

h Calculated by weighting ion score (based on the probability that ion fragmentation matches are non-random) events for all individual peptides matched to the protein. Ion scores for duplicated matches are excluded from the calculation.

i Confidence level of the total ion score.

j Calculated by Mascot search (−10×Log*P*) for identified proteins.

k Percentage of coverage of the identified proteins.

l Probability that the peptide mass matches are non-random events.

In the embryo, almost all proteins involved in development, defense, transport facilitation, cellular biogenesis, nitrogen metabolism, and lipid metabolism were up-regulated from 17 DAP to 25 or 28 DAP. Notably, most development-related proteins were late embryogenesis abundant (LEA) proteins and defense-related proteins comprised redox homeostasis regulation or antifungal proteins (Table 2).

### Transcriptional Profile of Protein sdh1 (Sorbitol Dehydrogenase Homolog 1, Spot no. 11)

**Figure 5:**
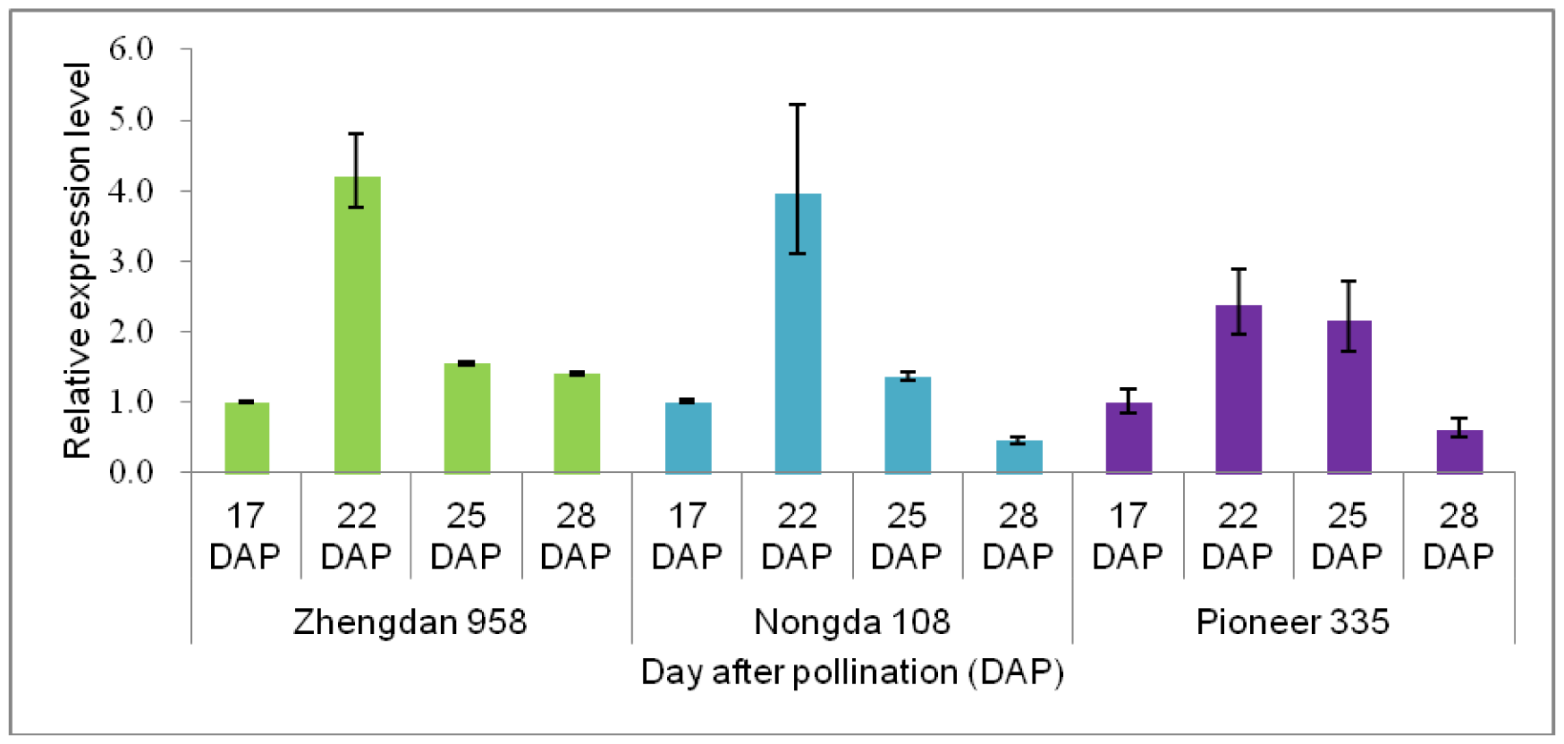
Relative expression level of the gene that encodes protein sdh1(spot no. 11).

Among the protein spots identified by MS, protein sdh1 was continuously up-regulated and showed the greatest difference in accumulation levels of all proteins–a more than 50-fold increase in Zhengdan 958 from 17 to 28 DAP. The transcriptional profile of the gene encoding this protein was assayed to explore the potential molecular mechanism at the transcriptional level. A similar pattern, with the expression peak at 22 DAP, was observed in each of the hybrids (Figure 5). In addition, the target sites of miRNA168 were identified in the coding gene with PlantPAN (http://plantpan.mbc.nctu.edu.tw/gene_group/index.php) programme.

### Integration with Genomic Information

To validate the potential implications of these functional annotations, the present results and those of a previous genetic analysis [20] were integrated for grain-filling rate (Table S3). Seven endosperm proteins, including one GenBank accession (gi|195618512) identified twice in the present investigation, were mapped to five QTL intervals on chromosomes (Chr.) 1, 2, 7, and 9. Nine distinct embryo proteins were mapped to three QTL intervals on Chr. 3, 8, and 9. Protein spot no. 11 was co-localized with a QTL for grain yield (*qGY1b*) [20] on Chr. 1 between the SSR markers umc1396 and umc1122.

## Discussion

### Key Period of Grain Filling

Grain yield is the result of accumulation of carbohydrates from leaf photosynthates during grain filling and non-structural carbohydrates in culms and leaf sheaths [22], [23]. Previous studies have demonstrated the importance of the former during the linear phase of grain filling for final yield [16]. In cereal crops, the effective and compensatory behavior of the duration and rate of grain filling influence the accumulation in the grain of carbohydrates from leaf photosynthates. Different from grain-filling duration, the grain-filling rate is positively correlated with, and critical for, the final grain yield [3], [4], [20], [23]. Therefore, the rate of carbohydrate transportation during the linear phase of grain filling has a fundamental effect on final grain yield based on the particular grain volume formed during the lag phase. Regardless of the genetic background, the peak grain-filling rate in maize inbred lines and hybrids occurs between 21 and 25 DAP [4], [20]. In the present study, the peak grain-filling rate was observed at 25 DAP for the three tested hybrids (Figure 1A). On the basis of changes in the grain-filling rate for the tested hybrids and data from a previous study [20], the critical period that influences dry matter accumulation was determined to be from 17 to 28 DAP. Four sampling stages were assayed both for the endosperm and embryo of each hybrid by 2-D gel electrophoresis in conjunction with MS identification, which identified some key factors that influence the carbohydrate transportation to the sink tissue (Table 2). Among the identified proteins, many were annotated as the ones involved in metabolic pathways, which implied to be important contributors to the grain filling rate and final yield. Exploration of the expression pattern and function of these proteins during the linear phase of grain filling should shed light on the genetic mechanism of kernel dry matter accumulation and contribute to understanding the regulation of grain development. The expression profiling for gene encoding sorbitol dehydrogenase homolog 1 implied that a specific signal might induce the high expression of this gene at 22 DAP (Figure 5).

### The Remobilization of Nutrients during Grain Filling Course

Two phases are involved in maize grain filling: 1) the production of carbon and nitrogen from carbohydrates, fats and proteins derived from photosynthetic and/or non-photosynthetic organs; 2) the synthesis and accumulation of the three nutrients-carbohydrates, fats and proteins in endosperm and embryo. This remobilization of nutrients would directly influence the grain filling rate and dry weight in different hybrids involves. Distinct metabolic processes should be integrated into the remobilization of nutrients, which requires a coordinating protein network [24].

In maize kernels, carbohydrates such as glucose, fructose and glutamine, as sources of carbon and nitrogen [25], are used by the endosperm and embryo to generate and accumulate different biomass products, which requires that these two tissues have different patterns of metabolism. Transportation of carbohydrates is a complicated process, and the synthesis of biomass products both in the endosperm and embryo requires several metabolic pathways to produce ATP and reducing power. Glycolysis/Gluconeogenesis pathway located in the cytoplasm of the cell is important for glucose conversion into pyruvate/the regeneration of a carbon source. The former is accompanied by the release of free energy that is used to form the high-energy compounds ATP and NADH. The enzymes involved in glycolysis pathway determine the dynamic balance of metabolites. Triose-phosphate isomerase plays a central role in glycolysis and influences efficient energy production, and has been identified in numerous plant studies [17]–[19], [26]–[29]. Triose-phosphate isomerase was identified in the endosperm of Zhengdan 958 (spot no. 17), Nongda 108 (no. 27), and Pioneer 335 (no. 39), which had different expression patterns. Glyceraldehyde-3-phosphate dehydrogenase, which functions in the sixth step of glycolysis to break down glucose for energy and carbon molecules, was also identified in the endosperm of Zhengdan 958 (spot no. 7) and Nongda 108 (no. 25), showing continuously up-regulated expression during grain filling course. This protein was identified only in the embryo of Zhengdan 958 with the least accumulation level at 22 DAP (Table 2, Table S1). Malate dehydrogenase involved in gluconeogenesis [30] is important for the generation of malate which is used to get oxaloacetate for tricarboxylic acid (TCA) cycle in the mitochondria. Its importance for the oxidation of malate to oxaloacetate might partially explain the identification in the endosperm and embryo. The location of the above mentioned proteins in the Glycolysis/Gluconeogenesis pathway (KEGG pathway *No.* zma00010) might reflect the importance of differential accumulation of Glycolysis/Gluconeogenesis related proteins during endosperm and embryo development. And the fact that these proteins were detected from different hybrids suggested that the differential regulation of Glycolysis/Gluconeogenesis pathway depended on the demand level for various metabolic intermediates of Glycolysis/Gluconeogenesis pathway during the course of kernel development in different hybrids. Pyruvate produced during glycolysis is used to provide precursors for biosynthesis of certain amino acids and the reducing agent NADH for the oxidative phosphorylation pathway to produce ATP energy in the TCA cycle. The TCA cycle is important for energy generation through the oxidation of the three nutrients-carbohydrates, fats and proteins and for precursors production of certain amino acids and reducing agent NADH, but only malate dehydrogenase, an enzyme involved in the regeneration of oxaloacetate, was identified with continuous down-regulation in the endosperm of Pioneer 335 (spot no. 32) and in the embryo of Nongda 108 with peak at 22 DAP (spot no. 59) in this study (Table 2). The mitochondrial localization of the reactions might be one possible reason for the small proportion of identified enzymatic proteins in the TCA cycle [17], [18]. The extreme importance of TCA cycle for the whole development of each organ might be another reason for the small proportion of differentially expressed proteins in this reaction during the sampling stages. The down-regulation expression of this protein was consistent with a great decrease in TCA cycle activity at later stages of development in the embryo and endosperm of this study, the embryo of maize [31], and the seed of Arabidopsis [32].

Based on the sources of carbon and nitrogen, carbohydrates, fats and proteins are re-synthesized as storage substances both in sink tissues, such as the endosperm, and in viable tissues, such as the embryo, and grain weight is added. Proteins control almost every reaction within a cell, provide structure and serve as signals to other cells. Thus, protein synthesis, including initiation, elongation, and termination steps, should have an important regulatory function in seed development. During the development process, a translation initiation factor is required for the correct assembly of an initiation complex and an elongation factor is required for effective elongation. Translation initiation factor 5A (spot no. 36) and translation elongation factor (spot no. 28) were identified in the endosperm and displayed down- and up-regulation expression trends, respectively, over the course of the grain-filling process in the present study (Table 2, Table S1, Figure 4). Translation initiation factors and elongation factors are the actual cellular effectors of protein synthesis, and their abundance, in turn, depends on the balance between protein translation and protein degradation. Protein-binding proteins that function to ensure correct protein folding and degradation were also identified in the endosperm of Zhengdan 958 (spot no. 2), Nongda 108 (spot nos. 19 and 21), and Pioneer 335 (spot no. 29) and were continuously up-regulated or peaked at 25 DAP. However, none of the protein synthesis-related proteins was identified in the embryo of the tested hybrids. The difference might be attributable to the distinct developmental requirement of the endosperm and embryo during the linear phase of grain filling. This conclusion is supported by the accumulation of storage proteins in the embryo (spot nos. 52, 78, and 79). The storage proteins globulin 1 (spot no. 78) and 2 (spot nos. 52 and 79), which are destinations of remobilized nitrogen and sulfur [33], were up-regulated during embryo development, which suggested globulin modulated efficient partitioning of nutrients during the linear phase of grain filling. The level of storage proteins, a destination of accumulated nutrients, may contribute to grain-filling rate and grain weight. Proteins involved in lipid metabolism were represented by different protein spots (nos. 50, 65, and 74) but showed a similar up-regulated expression pattern (Table S1) in the three hybrids. Meanwhile, proteins involved in nitrogen metabolism were detected with different expression patterns in Nongda 108 (spot nos. 62 and 67) and Pioneer 335 (spot no. 75) (Table S1). Phytic acid is a phosphorylated derivative of myo-inositol, functions as the major storage form of phosphorus in plant seeds, and plays diverse roles in plants as a signal transduction molecule, osmoprotectant, and cell wall constituent. The inositol-3-phosphate synthase (spot no. 71) identified in the present study catalyzes the first step in *de novo* synthesis of myo-inositol. This protein showed continuous down-regulation, opposite to the expression of proteins involved in lipid metabolism and nitrogen metabolism, in Pioneer 335 (spot no. 71), which supported its negative regulation of grain-filling rate during the linear phase.

### Specific Grain Filling Related Proteins in Embryo and Endosperm

Embryo development represents a critical stage in the sporophytic life cycle. The embryo becomes desiccated and dormant at late stages of development [34]–[36]. A group of hydrophilic proteins, known as LEA proteins, accumulate to high levels during the final stage of seed maturation [37]. These proteins are neither enzymes nor true storage proteins, but probably serve to protect embryos from desiccation stress and stabilize other proteins and membranes that protect the cell [38]–[40]. The genes that encode LEA proteins belong to a multigene family [40], [41]. The protein LEA 34 identified in the present study (spot nos. 51, 64, and 76) belongs to LEA group 5, of which transcripts accumulate during late stages of seed development and in response to drought [42], [43]. Generally, LEA proteins accumulate in embryos soon after storage protein accumulation and their synthesis in embryos coincides with desiccation of the seed. LEA 34 was identified in the embryo of the three hybrids and showed similar up-regulated expression during seed development. An additional embryo-abundant protein (spot no. 68) and embryo-specific protein (spot no. 80) identified in this study also showed up-regulated expression during seed development. These proteins may represent markers for embryo development stages [44].

The endosperm, which is an important nutritional supply tissue for embryo development during seed germination, should accumulate abundant starch during the linear phase of grain filling. However, none of the enzymes involved in the starch synthesis pathway was identified in the present study. Three possible reasons might explain this result: 1) differential expression of enzymes involved in starch synthesis was less than 2-fold during the linear phase of grain filling; or 2) starch synthesis, in which ADP glucose is converted to amylose or amylopectin by starch synthases and starch-branching enzyme, does not have a strong and direct regulatory relationship with sucrose transportation in the cytosol controlled by grain-filling rate during the linear phase; or 3) the quantitative sensitivity limitations of the technology used in this study might also be a possible reason. Sorbitol dehydrogenase is an enzyme in carbohydrate metabolism converting sorbitol, the sugar alcohol form of glucose, into fructose [45]. The spot no. 11 identified in this study is a sorbitol dehydrogenase homolog 1 protein, which was continuously up-regulated with the greatest difference in accumulation levels of all proteins and expressed a peak at 22 DAP at the transcriptional level for all hybrids tested here (Figure 5). Integration with genomic information verified that its coding gene was co-localized with a QTL for grain yield (*qGY1b*) on Chr. 1 [20]. In addition, the target sites of miRNA168 were also identified in this gene. These results implied that sorbitol dehydrogenase gene regulated by miRNA might be an important regulator of maize grain filling, especially for hybrid Zhengdan 958.

## Materials and Methods

### Plant Material

Three elite maize hybrids that show significant differences in grain-filling rate and grain-filling duration were used in this study. Nongda 108 (Xu 178×Huang C) was the most widely planted hybrid (in terms of planting area) between 2002 and 2004 in China, Zhengdan 958 (Zheng 58×Chang 7-2) was the most widely planted hybrid between 2005 and 2012 in China, and Pioneer 335 had the third-largest planting area between 2008 and 2012 in China. To obtain a similar flowering time in the field, Nongda 108 was planted on 1 May 2011, and Zhengdan 958 and Pioneer 335 were planted on 5 May 2011, which resulted in a flowering time between 4 and 9 July. For each genotype, two replication plots were planted on the research farm of Henan Agricultural University, Zhengzhou, China (113°42′ E, 34°48′ N). The plots consisted of eight rows of 4 m length, with inter-row spacing of 75 cm and within-row spacing of 25 cm. The hybrids were self-pollinated by hand on 6 July and ears were collected from each plot at 10, 17, 22, 25, 28, 33, 40, and 50 DAP. Only the middle rows were sampled to avoid edge effects. Kernels from the middle part of 90 ears were separated into two groups for each plot. Three one-hundred kernels per replication plot were oven-dried at 70°C for 24 h and the dry weight was recorded. The final dry weight of each replication plot was calculated by averaging the three 100-kernel dry weight values. The grain-filling rate was calculated by dividing the dry matter accumulated by the number of days between two sampling stages. The remainder of the kernels was dissected to remove the pericarp, and the endosperm and embryo were dissected for three biological replicates and stored at –80°C until use for protein and RNA extraction, respectively. Data analysis was performed using the SAS 8.0 statistical software package with the PROC MIXED procedure. On the basis of the change in grain-filling rate during the sampling stages (Figure 1A), endosperm and embryo samples harvested at 17, 22, 25, and 28 DAP for each genotype were subjected to further proteomic analysis.

### Protein Extraction

The three biological replicates per tissue sample on each sampling date per hybrid were prepared. Approximately 1 g for each sample was ground in liquid nitrogen. Ten milliliters of chilled trichloroacetate buffer containing 10% trichloroacetate and 0.07% β-mercaptoethanol (in acetone) was added and mixed in tube. After 2 h incubation at –20°C, the extracts were centrifuged at 18,000×*g* for 30 min at 4°C and the supernatant was discarded. Ten milliliters of chilled 80% acetone containing 0.07% β-mercaptoethanol was added to each sample, mixed well and incubated for 1 h at –20°C before centrifuging at 18,000×*g* for 15 min at 4°C. This step was repeated twice and the pellet was freeze-dried under vacuum for embryo samples. The pellet for endosperm samples was added the following steps for the starchy characteristics. The pellet was resuspended in extraction buffer (30% sucrose, 0.1 M Tris-HCl [pH 8.0], 1% 1,4-dithio-DL-threitol [DTT], 100 mM KCl, 5 mM EDTA, 2 mM phenylmethylsulfonyl fluoride). The mixture was centrifuged at 18,000×*g* for 10 min at 4°C. The final combined supernatant was transferred to a new tube, and an equal volume of Tris-phenol was added and mixed. After centrifugation at 18,000×*g* for 30 min at 4°C, the phenol phase and a 5-fold volume of chilled 0.1 M ammonium acetate (in methanol) were incubated at –20°C for 30 min. The supernatant was discarded, and the protein pellet was collected by centrifugation at 12,000×*g* at 4°C for 30 min. After two thorough washes with cooled 0.1 M ammonium acetate (in methanol) and one wash with 80% cooled acetone, the resulting proteins were freeze-dried under vacuum. A solution containing 9 M urea, 2 M thiourea, 4% (w/v) CHAPS, and 1% DTT was added to the dried protein pellets at a ratio of 20 µl mg^−1^. After incubation for 1 h at 28°C with frequent shaking, the insoluble fraction was removed via centrifugation at 18,000×*g* for 40 min at 25°C. The concentration of the supernatant containing the soluble protein fraction was determined by the Bradford method with bovine serum albumin used as a standard [46].

### Two-dimensional Gel Electrophoresis

The final extracted proteins were subjected immediately to 2-D gel electrophoresis or stored in aliquots at −80°C. Isoelectric focusing (IEF) of the proteins for each biological replicate of each genotype was performed with three technical replicates. For each genotype, samples of the four different developmental stages were analyzed simultaneously for convenience of comparison. For each replication, equal amounts of total protein extract (1 mg) were assayed using 24 cm immobilized dry strips (Immobiline DryStrips, Bio-Rad, Hercules, CA, USA) with a linear pH gradient of pH 4–7. After rehydration for 11 h at 50 V, IEF was performed at 18°C with the following conditions: 200 V for 1 h, 500 V for 1 h, 1000 V for 1 h, 5000 V for 1 h, and 10,000 V for 10 h. Current was limited to 0.05 mA per immobilized pH gradient gel strip. Equilibration of the strips was performed immediately with 10 mL of two types of sodium dodecyl sulfate (SDS) equilibration buffer for 15 min each. Buffer 1 contained 0.375 M Tris-HCl (pH 8.8), 6 M urea, 20% glycerol, 4% SDS, and 2% DTT, and buffer 2 contained 0.375 M Tris-HCl (pH 8.8), 6 M urea, 20% glycerol, 4% SDS, and 2.5% iodoacetamide. The immobilized pH gradient gel strips with the proteins were embedded in the top of a 2-D gel in 1% agarose solution after equilibration. Proteins were separated on the basis of their *M*_r_ on a 12% SDS-PAGE gel at 16°C under the following conditions: 1 W of constant power for 30 min, then increased to 6 W, which was maintained until the electrophoresis was finished. Preparative gels were stained with Coomassie Brilliant Blue R250 and imaged by a laser scanner (AlphaImager HP, Alpha Innotech, Santa Clara, CA, USA).

### Identification of Protein Spots by Mass Spectrometry

Only protein spots that appeared consistently in nine technical replicates of the three biological replicates for each genotype were counted. Their expression was analyzed using Imagemaster 2-D Platinum software version 5.0 (Bio Rad). Only protein spots with more than 2-fold changes between the maximum expression level and the lowest expression level (absolutely intense difference) at different DAP stages for each genotype, as determined by ANOVA and Student’s *t*-test (*P*<0.01) after normalization, were excised manually and processed for MS. Spots from the 2-D gel were transferred individually to a microcentrifuge tube (Eppendorff) and destained with 25 mM NH_4_HCO_3_ in 50% (v/v) acetonitrile for three periods of 15 min each at room temperature. Gel spots were dried using a vacuum centrifuge at room temperature and incubated in 50 µl digestion solution, which consisted of 25 mM NH_4_HCO_3_ in 0.1% acetic acid and 12.5 ng mL^−1^ sequencing-grade trypsin at 37°C overnight (Promega, Madison, WI, USA). After digestion, a 0.3 µl sample was spotted onto a matrix-assisted laser desorption/ionization time-of-flight mass spectrometry (MALDI-TOF) sample plate with the same volume of matrix (10 mg mL^−1^ α-cyano-4-hydroxycinnamic acid in 50% acetonitrile, 0.1% trifluoroacetic acid). Peptide mass spectra were obtained on a MALDI-TOF/TOF mass spectrometer (4700 Proteomics Analyzer, Applied Biosystems) in the positive ion reflector mode. The MS spectra were calibrated internally with the Mass Standards kit for the 4700 Proteomics Analyzer. Proteins were identified by automated peptide mass fingerprinting using the Global Proteome Server Explorer™ software version 3.5 (Applied Biosystems) against maize protein FASTA sequences (94,845 sequences) in the NCBI database. The unknown and unnamed protein spots were further searched in the KEGG database with blast function. Peak lists (*S*/*N* >10) were extracted from the raw data for data processing, and positive identifications were accepted up to the 95% confidence level using the following criteria: 1) precursor ion mass tolerance ±0.1 Da; 2) fragment ion mass tolerance ±0.3 Da; 3) maximum of one missed cleavage per peptide allowed; and 4) fixed modifications by cysteine carboxyamidomethylation and variable modifications by methionine oxidation.

### Transcript Profiling

Endosperm tissue at 17, 22, 25, and 28 DAP were assayed for transcript profiling in accordance with the protein sampling date. Three samples were prepared on each sampling date per hybrid. Total RNA was isolated using the Plant Total RNA Extraction kit (Bioteke Corporation, China) in accordance with the manufacturer’s protocol. Absence of genomic DNA contamination was confirmed by PCR, using primers designed to flank the intron sequences of a control gene (Actin: 5′-TGGCATTGTCAACAACTGG-3′, 5′-CTCCTTGCTCATACGATCGG-3′). First-strand cDNA was synthesized using oligo (dT)15 as a primer and M-MLV Reverse Transcriptase (Promega, USA) in a 25-µl reaction volume. Quantitative real-time PCR was performed using SYBR® Premix Ex Taq™ (Takara, Dalian, China) on a DNA Engine Option®2 Continuous Fluorescence detection system (Bio-Rad, Waltham, USA). Melting curve analysis was performed to verify primer specificity. The relative quantity of the transcripts was calculated using the comparative threshold cycle method. An *Actin* control was used to normalize data across samples. Each sample was analyzed in triplicate for each gene and *Actin*, and the average value for each was used in analyses. The sequences of *Actin* primers for real-time PCR were 5′-CGATTGAGCATGGCATTGTCA-3′ and 5′-CCCACTAGCGTACAACGAA-3′.

## Supporting Information

Figure S1 **2-D maps of the maize hybrids Nongda 108 and Pioneer 335.** From left to right, 17, 22, 25, and 28 DAP are presented. Differentially expressed protein spots at any two stages with more than 2-fold changes in expression are indicated by arrows with numeral. (A) 2-D maps for endosperm of Nongda 108. (B) 2-D maps for endosperm of Pioneer 335. (C) 2-D maps for embryo of Nongda 108. (D) 2-D maps for embryo of Pioneer 335.
(TIF)

Figure S2 **Changing pattern classification of differentially expressed proteins identified by MS at sampling stages.** I, Proteins whose abundance increased linearly from 17 to 28 DAP; II, proteins whose abundance decreased linearly from 17 to 28 DAP; III, proteins up-regulated at 25 DAP; IV, proteins down-regulated at 25 DAP; V, proteins up-regulated at 22 DAP; VI, proteins down-regulated at 22 DAP; VII, proteins that changed irregularly.
(TIF)

Table S1 **Expression levels of differential proteins identified by MS at sampling stages.** I, Proteins whose abundance increased linearly from 17 to 28 DAP; II, proteins whose abundance decreased linearly from 17 to 28 DAP; III, proteins up-regulated at 25 DAP; IV, proteins down-regulated at 25 DAP; V, proteins up-regulated at 22 DAP; VI, proteins down-regulated at 22 DAP; VII, proteins that changed irregularly.
(XLS)

Table S2 **Functional annotation of differentially expressed proteins identified by MS.** Repeated peptides were omitted.
(XLS)

Table S3 **Integration of QTL intervals and differentially expressed proteins identified by MS for grain-filling rate.**
(XLS)
