# Supplementary figures and images for: Proteomic Identification of Genes Associated with Maize Grain-Filling Rate
Source: PLoS One. 2013 Mar 19;8(3):e59353. doi: 10.1371/journal.pone.0059353 (PMC3601958; doi:10.1371/journal.pone.0059353)

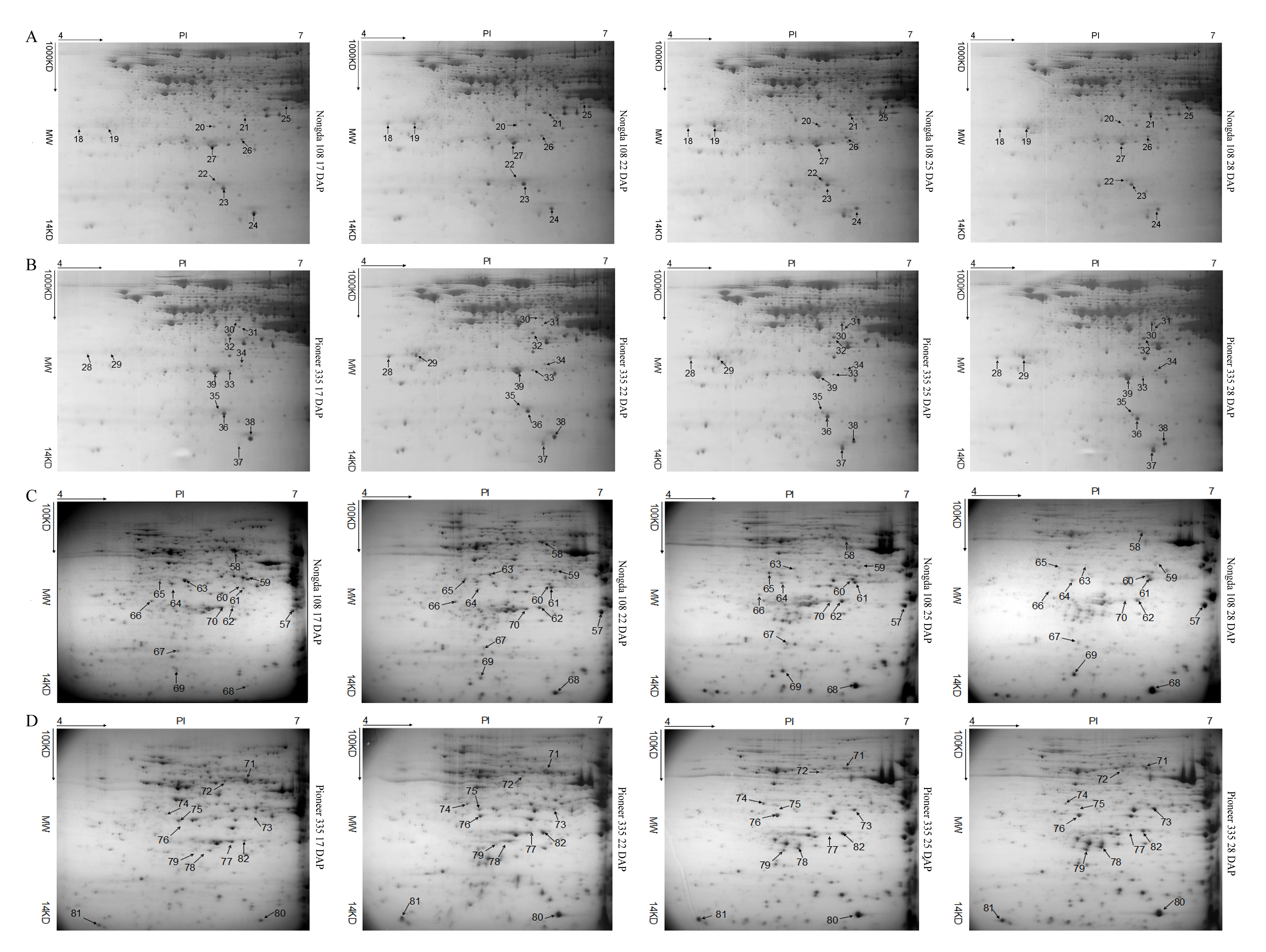

Supplement: Figure S1 — 2-D maps of the maize hybrids Nongda 108 and Pioneer 335. From left to right, 17, 22, 25, and 28 DAP are presented. Differentially expressed protein spots at any two stages with more than 2-fold changes in expression are indicated by arrows with numeral. (A) 2-D maps for endosperm of Nongda 108. (B) 2-D maps for endosperm of Pioneer 335. (C) 2-D maps for embryo of Nongda 108. (D) 2-D maps for embryo of Pioneer 335. (TIF) [file pone.0059353.s001.tif]

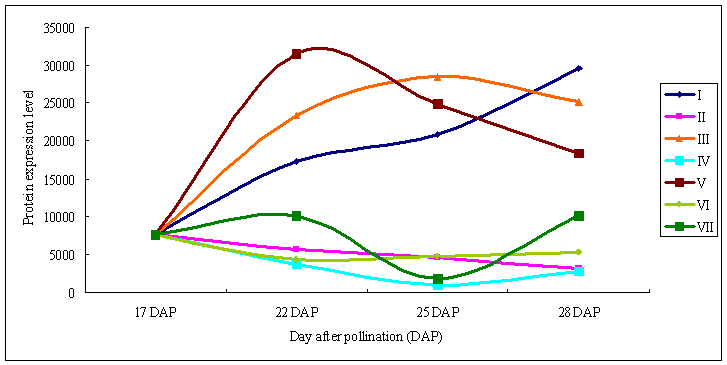

Supplement: Figure S2 — Changing pattern classification of differentially expressed proteins identified by MS at sampling stages. I, Proteins whose abundance increased linearly from 17 to 28 DAP; II, proteins whose abundance decreased linearly from 17 to 28 DAP; III, proteins up-regulated at 25 DAP; IV, proteins down-regulated at 25 DAP; V, proteins up-regulated at 22 DAP; VI, proteins down-regulated at 22 DAP; VII, proteins that changed irregularly. (TIF) [file pone.0059353.s002.tif]
